# Promoting Electroreduction CO_2_ to Multi‐Carbon Products by Tailoring the ^*^H Availability With Optimized Interfacial H_2_O Configuration

**DOI:** 10.1002/advs.76470

**Published:** 2026-07-17

**Authors:** Dawei Zhou, Songhu Bi, Jie Zhang, Jinghui Deng, Keyi Xu, Lin Xia

**Affiliations:** ^1^ Key Laboratory of Quantitative Synthetic Biology Shenzhen Institute of Synthetic Biology Shenzhen Institute of Advanced Technology Chinese Academy of Science Shenzhen China; ^2^ Shenzhen Powered Carbon Biotechnology Co., Ltd. Shenzhen China

**Keywords:** carbon dioxide, C_2+_ products, electrocatalysis, ionomer, interfacial H_2_O configuration

## Abstract

The electroreduction of CO_2_ to multi‐carbon products provides a promising path for approaching carbon neutrality. H_2_O is generally considered a hydrogen source in CO_2_RR, tailoring the interfacial H_2_O configuration is critical yet often overlooked strategy for modulating CO_2_RR performance. In this work, we demonstrated that regulating the interfacial water configuration through ionomer confinement is an effective strategy for enhancing multi‐carbon (C_2+_) product selectivity in CO_2_RR. The optimized catalyst 15.79% A5‐Cu achieves 82% Faradaic efficiency (FE) for C_2+_ products at 200 mA cm^−2^ in 0.1 M KHCO_3_ and operates stably for over 100 h without salt precipitation in MEA system. In situ ATR‐SEIRAS and Raman spectroscopy revealed that the confined environment tunes the proportion between K^+^‐H_2_O and 4‐HB‐H_2_O configuration of interfacial H_2_O. This enhanced *H generation and transfer ability can improve the *H utilization efficiency. Moreover, a moderate *H availability constructed by the balanced proportion of K^+^‐H_2_O and 4‐HB‐H_2_O configurations lowers the energy barrier for *CO hydrogenation and subsequently C─C coupling step at a higher *CO coverage, which was confirmed by DFT calculations. This work highlights the critical role of interfacial H_2_O configuration in steering C_2+_ product selectivity for that in addition to *H generation, the hydrogen bonding network can accurately guide *H toward *CO intermediate, thereby avoiding excessive accumulation of *H species.

## Graphical abstract

A catalysis system with adjustable interfacial H_2_O configuration was conducted by introducing ionomer into Cu. The increasing proportion of K^+^‐H_2_O can enhance the dissociation of H_2_O and improve the generation ability of *H, and the increasing proportion of 4‐HB‐H_2_O can accelerate the transfer speed of *H to *CO intermediates with the aid of strong hydrogen bonding network, the synergistic effect of K^+^‐H_2_O and 4‐HB‐H_2_O configurations can precisely guide proton flow toward the C_2+_ products pathway, effectively suppressed the HER.

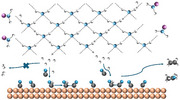

## 1 Introduction

Electrochemical CO_2_ reduction reaction (CO_2_RR) powering by the renewable energy has been considered as an attractive path for approaching the carbon neutrality [1, 2, 3, 4]. The product selectivity toward multi‐carbon (C_2+_) hydrocarbons, such as ethylene, ethanol and propanol of Cu‐based catalysts is widely concerned due to their potential market application value and energy density. Up to now, most studies about CO_2_RR have mainly focused on the rational design in catalysts [5, 6, 7, 8]. However, H_2_O‐containing electrolytes, which provide a medium for electron and proton transfers, also significantly affect the activity and selectivity for CO_2_RR. Interfacial H_2_O molecules, serving not only as proton source for the hydrogenation process of CO_2_ reduction intermediates but also for the competitive hydrogen evolution reaction (HER) [9, 10]. Generally, the relative coverage of key species (such as *H and *CO) on Cu surface determines reaction direction toward C_1_ or C_2+_ products, and maintaining an appropriate *H coverage and increasing *CO coverage is a universal strategy for promoting the selectivity of C_2+_ products [11, 12]. Since the generation of *H originating from the dissociation of interfacial H_2_O, we can reasonably propose that tailoring the properties of interfacial H_2_O, may effectively regulate the rate variability of *H supplied to the surface active sites and thereby controlling the CO_2_RR product selectivity [13, 14, 15].

During the process of CO_2_RR to C_2+_ products, in addition to the C─C coupling steps, another critical but often overlooked is the *H‐involved proton‐coupled electron transfer (PCET) and hydrogenation of *CO intermediate process, which will undergo C─C coupling to generate C_2+_ products [16, 17]. In cation‐containing electrolyte, cation absorption behavior, which results in *CO intermediate enrichment [18], the change of local pH [19], interfacial electric field modulation [20] and intermediate stabilization [21, 22] was mainly used to explain the promoted CO_2_RR performance; however, recently Hall and co‐workers found that in low cation strengths electrolyte, the cation absorption plays a role in regulating *CO surface coverage, but high concentration electrolytes promote the shift toward C_2+_ products by altering the interfacial H_2_O structure, rather than through cation coverage [23]. So, to re‐examine the multi‐functionality of interfacial H_2_O characteristic in cation‐containing electrolyte, further explore should continue.

**SCHEME 1:**
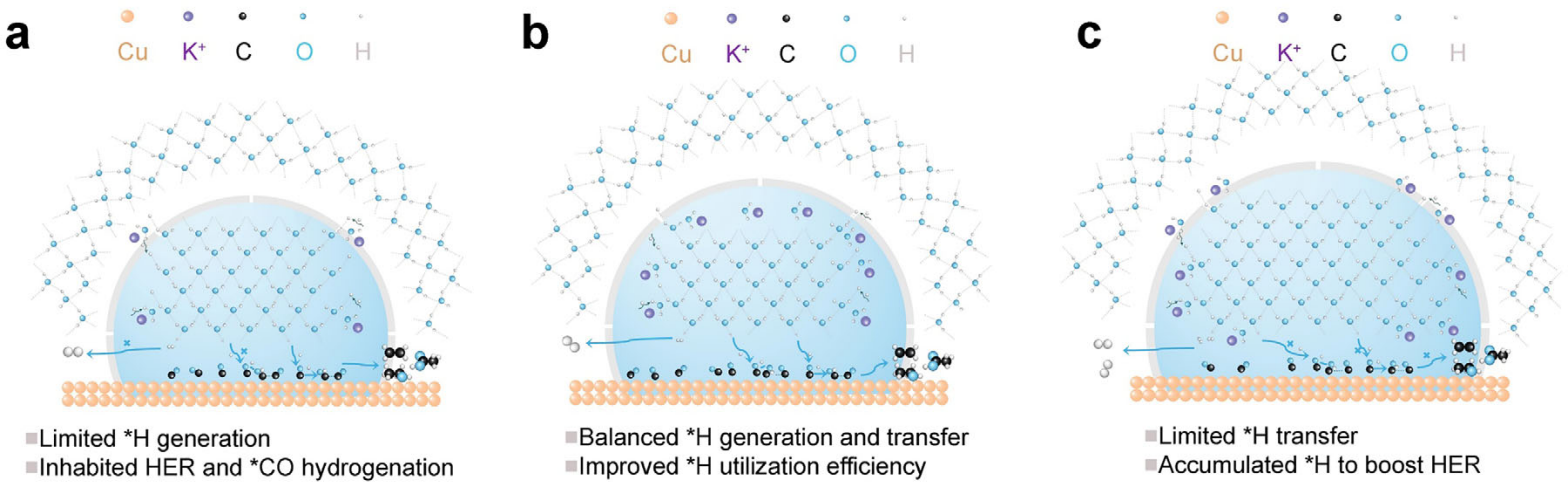
The schematic diagram of proposed *H microenvironment in CO_2_RR. (a) The limited *H generation. (b) The balanced *H generation and transfer. (c) The limited *H transfer.

In fact, the configuration of interfacial H_2_O is closely related to its located microenvironment [24]. For example, strongly hydrogen‐bonded H_2_O with a certain rigidity requires an unrestricted space area to extend the robust hydrogen bonding network, and higher cation strength is beneficial for the formation of ionized H_2_O. Considering the involvement of *H species in CO_2_RR, ionized H_2_O is more easily to release *H through H_2_O dissociation, the generation *H can transfer to *CO intermediates through hydrogen bonding network of strong hydrogen bonded H_2_O [25, 26, 27]. Matching the process of *H generation and transfer, maintaining optimized proton utilization efficiency, is crucial for promoting the selectivity of C_2+_ products (the proposed schematic diagram was shown in Scheme 1a–c). Obviously, the *H species participating in CO_2_RR usually contain two aspects. However, considerable researches ignored the synergistic effect of *H generation and *H transfer process, this overlook may also constrict CO_2_RR performance [28, 29, 30].

**FIGURE 1:**
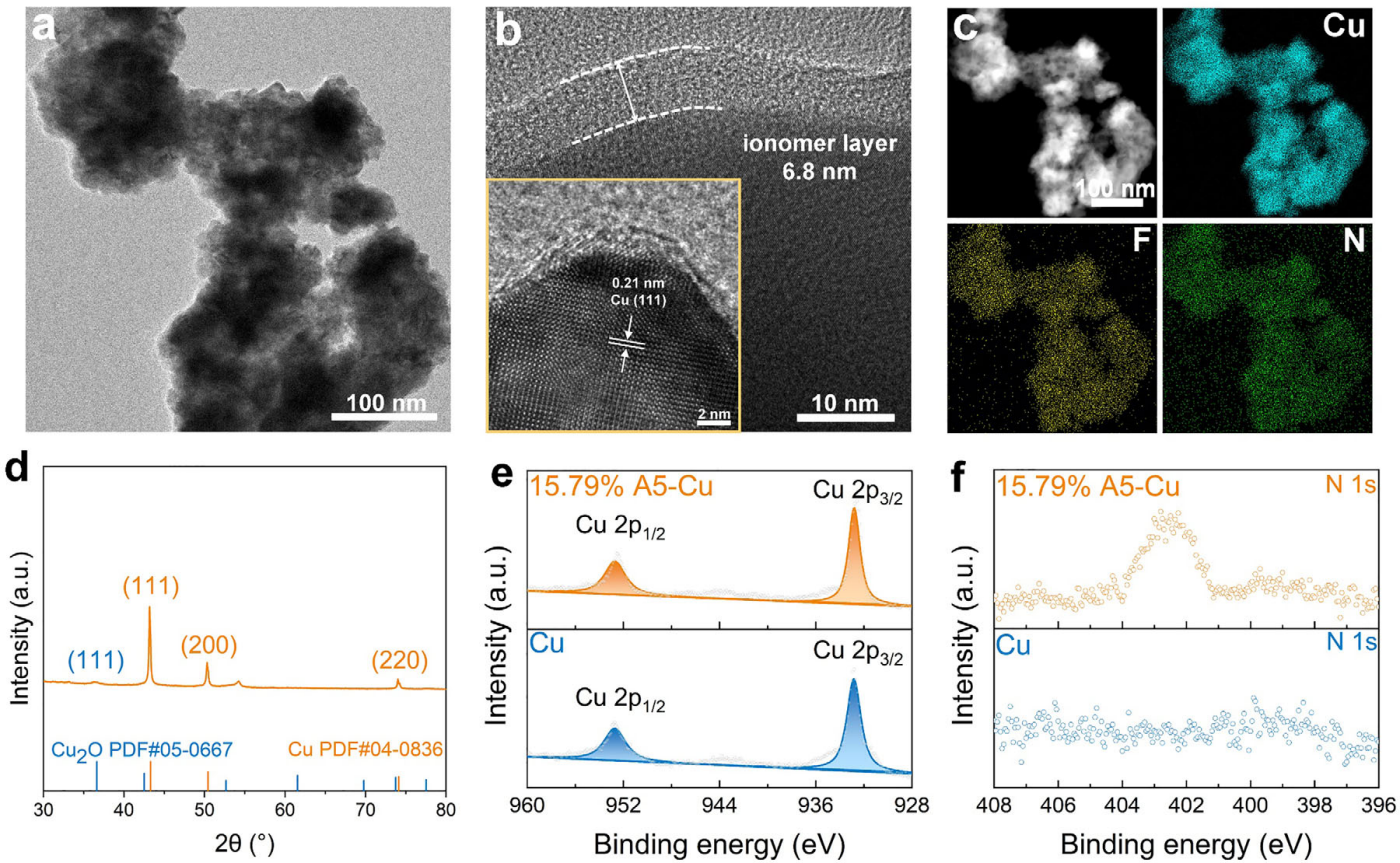
(a) The structural and surface valence state characterization of catalysts. (a) TEM image of the 15.79% A5‐Cu catalyst. (b) HR‐TEM image of 15.79% A5‐Cu catalyst. (c) EDS element mapping images of the 15.79% A5‐Cu catalyst. (d) XRD pattern of the 15.79% A5‐Cu catalyst. (e) Cu 2p XPS spectra of Cu and the 15.79% A5‐Cu catalysts. (f) N 1s XPS spectra of Cu and the 15.79% A5‐Cu catalysts.

In most reported cases, the authors tailor the *H availability by constructing multi‐catalytic sites [31, 32, 33, 34] and fabricating built‐in electric field [17], or tuning interfacial H_2_O orientation [35, 36] to lower the energy barrier of *H generation for promoting H_2_O dissociation. In addition, for the ionomer‐involved condition, the enhanced interfacial hydrophobicity was usually used to explain the *H availability for that a proper *H microenvironment can greatly improve the CO_2_RR performance. However, these studies did not explicitly decouple the *H transfer from the *H generation process, nor did they quantify its contribution to *H availability (Scheme 1). Accurately constructing a spatial network for the generation and transfer of *H species, optimizing migration paths, is a promising strategy. Recent studies have shown that interfacial H_2_O configuration can be modified by introducing organic ionomer on the surface of catalysts. For Cu‐based catalysts, the improved selectivity toward the C_2+_ products usually ascribed to the increased hydrophobicity, enhanced CO_2_ local concentration in the catalyst layer or cation enrichment at the interface [37, 38]. Actually, the introduction of ionomer not only preserves the channel for mass transport, but also affects the diffusion behavior of cations, especially in the regulation of interfacial H_2_O configuration due to spatial confinement effect. Therefore, creating an optimized confined microenvironment for efficient *H availability might be an emerging strategy to guide the design efficient CO_2_RR system by introducing ionomer [39, 40].

Herein, employing the PiperION‐A5 ionomer‐confined Cu catalyst, we investigated the effect of interfacial H_2_O configuration evolution on C_2+_ products selectivity during CO_2_RR. In situ ATR‐SEIRAS and Raman spectroscopy results suggested that confined effect induced by ionomer can significantly influence the proportion of interfacial H_2_O configuration. As the precursor for *H generation, increasing the proportion of K^+^‐H_2_O can improve the *H supplied ability for subsequent hydrogenation; as the *H space migration network, enhancing the proportion of 4‐HB‐H_2_O can accelerate the speed for *H species transfer to *CO intermediate. Combining with DFT calculation, we found that a balanced proportion of 4‐HB‐H_2_O and K^+^‐H_2_O configuration can markedly decrease the energy barrier of *CO hydrogenate to *COH, improve *H utilization efficiency to enhance the C_2+_ products selectivity at a higher *CO coverage. As a result, by optimizing the ionomer addition content in the PiperION‐A5 confined Cu catalyst, the FE of C_2+_ products could reach to 82% for at least 100 h at 200 mA cm^−2^ without salt precipitate in 0.1 M KHCO_3_. This research disclosed an often easily overlooked issue in *H‐involved CO_2_RR system that compared with the *H generation step, the transfer ability of *H species are equally critical in regulating the selectivity of C_2+_ products.

## 2 Results and Discussion

### 2.1 Catalysts Preparation and Characterization

To elucidate how the confined effect governs the C_2+_ products selectivity by tailoring the interfacial H_2_O configuration, we constructed a series of PiperION A‐5 ionomer‐modified Cu catalysts (wt.% A5‐Cu) to simulate confined microenvironments at different nanoscale through coordination‐precipitation‐annealing method followed by the in situ activation. In a typical synthesis, Cu(NO_3_)_2_ aqueous solution and ammonia aqueous solution were added to prepare the blue complex, then the blue precipitate of Cu(OH)_2_ was obtained when dropwise added of NaOH aqueous solution. After filtered washing and freeze‐drying, the nano CuO was prepared by annealing Cu(OH)_2_ in the air atmosphere. Next, the PiperION‐A5 anion exchange ionomer was added into the CuO disperse solution with ultrasound under the ice‐bath condition, and then the as‐prepared catalyst ink was sprayed on the GDL. Finally, the A5‐modified CuO GDL electrode was in situ electroreduction to form A5‐Cu catalyst (The corresponding denoted name can be seen in Table S1). The detailed information of this process is shown in the supporting information.

The XRD pattern results (Figure S1) showed the addition of A5 ionomer shares the same diffraction peaks with the CuO phase (PDF#44‐0706), Scanning electron microscopy (SEM) (Figure S2a) and transmission electron microscopy (TEM) images (Figure S3) revealed that the synthesized CuO exhibited a characteristic nanostructure with average particle size of 50–150 nm. After the in situ electroreduction, TEM images revealed the Cu catalyst still retained the nano characteristic of the CuO precursor (Figure S4), but the A5‐confined Cu catalysts exhibit a uniform rough surface with a thin ionomer layer covering the inside nano Cu (Figure 1a and Figures S5–S7a). High‐resolution TEM (HR‐TEM) image of 15.79% A5‐Cu catalysts had a lattice fringe spacing of 0.21 nm, consistent with the Cu (111) facet (Figure 1b). Element distribution mappings of Cu, F, and N were displayed in Figure 1c, confirmed the uniform distribution of ionomer on the surface of Cu. The structure, morphology, and element distribution of others catalysts were shown in Figures S5–S7. The thickness of ionomer layer varied depending on the addition content, for the 5.88%, 11.11%, 15.79%, and 20% A5‐Cu catalysts, the ionomer layer thickness was about 2.6, 4.7, 6.8, and 11.6 nm, respectively (Figure 1b and Figures S5–S7b). Moreover, as shown in Figure 1d, new peaks emerged at 43.3°, 50.4°, and 74.1° over 15.79% A5‐Cu catalyst, which were identical with the (111), (200), and (220) crystalline planes of metallic Cu (PDF#04‐0836), respectively. The weak peak located around 36.4° which belongs to the (111) facet of Cu_2_O may be resulted from the Cu oxidation during XRD detection. In addition, the XPS analysis was conducted to detect the valence state of catalyst surface species (Figure 1e,f and Figure S8). After in situ electroreduction, the disappearance of Cu^2+^ and satellite peaks is accompanied by the appearance of Cu^0^ peaks on the surface, and there is no significant change of the N 1s signal belonging to the A5. These characterization results indicated that the metallic Cu was the active site and the A5 ionomer could maintain relative stability during CO_2_RR.

### 2.2 CO_2_RR Performance

To verify the hypothesis we mentioned above, we evaluated the CO_2_RR performance between Cu and A5‐Cu catalyst in the MEA system with 0.1 M KHCO_3_ aqueous solution as electrolyte. The CO_2_RR experiments were conducted under the constant current mode on a DC power supply device, after every electrolysis procedure was finished, the collected gaseous and liquid products of CO_2_RR were analyzed by gas chromatography (GC) and ^1^H nuclear magnetic resonance (^1^H NMR) spectroscopy, respectively. The other experiment detail is listed in the supporting information.

**FIGURE 2:**
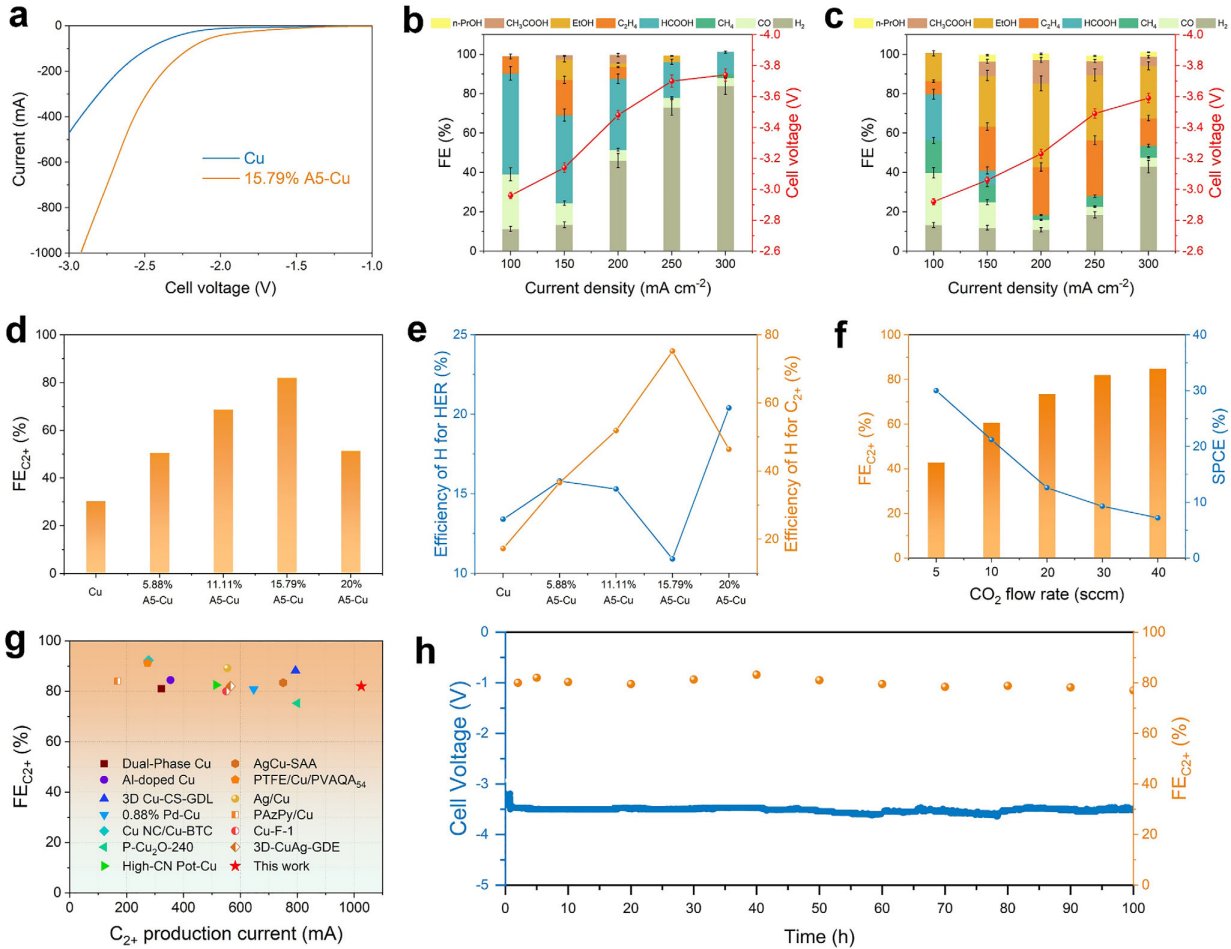
(a) The *I–V* curves of Cu and the 15.79% A5‐Cu catalysts. (b) The FE and applied cell voltages of Cu catalyst at different applied current density. (c) The FE and applied potentials of 15.79% A5‐Cu catalyst at different applied current density. (d) The optimized FE of C_2+_ in different A5 ionomer addition content (wt.%) Cu catalysts. (e) The H utilization efficiency for HER and C_2+_ products generation at 200 mA cm^−2^. (f) The plot of FE and SPCE of C_2+_ products varied with CO_2_ flow rate. (g) The performance comparison of this work with the state‐of‐the‐art results. (h) CO_2_RR long‐term stability of 15.79% A5‐Cu catalyst at a constant current density of 200 mA cm^−2^ in MEA system.

The *I–V* curves was first conducted to assess the catalytic performance in CO_2_‐saturated electrolytes. As shown in Figure 2a, 15.79% A5‐Cu demonstrated significantly higher current signal than the bare Cu catalyst. Then we began to evaluate the effect on CO_2_ performance of ionomer content, the maximal FE of C_2+_ products for the nano Cu catalyst was 30.3% at current density of 150 mA cm^−2^, as the addition content of ionomer gradually increases, the FE of C_2+_ products first increases and then decreases, reaching its maximum at 15.79% at 200 mA cm^−2^ (as shown in Figure 2b,c). Under the optimized condition, the FE of C_2+_ products could reach to 50.5%, 68.7%, 82%, and 51.3% for 5.88%, 11.11%, 15.79%, and 20% A5 modified Cu catalysts, respectively (Figure 2c and Figure S9). Comparing these optimized FE of C_2+_ products in Figure 2d and Figure S10, the results showed that a moderate A5 addition content can markedly enhance the C_2+_ products selectivity and C_2+_ partial current density. The H content in reduction products reflected the tendency of the catalytic system to obtain *H from H_2_O, we compared the *H utilization efficiency (The details of the calculation are in supporting information) between HER and CO_2_RR (Figure 2e). The results indicated that 15.79% A5‐Cu had the worst *H accepting ability for HER, on the contrary, the utilization efficiency of *H participating in the generation of C_2+_ products ranked its peak at 15.79% A5‐Cu. Moreover, as the reduction products required 2 and 8 proton‐coupled electron transfer (PCET) process respectively, the comparison of FE between HCOOH and CH_4_ was used to show the availability of *H for catalyst system. As shown in Figure S11, the FE of HCOOH was continuously decreased with ionomer content gradually increased, but the FE of CH_4_ was slightly increased from 0 to 3.7%, this indicated *H generation and transfer ability was dynamic. To evaluate the single‐pass carbon efficiency (SPCE), we changed the CO_2_ flow rate during CO_2_RR, the SPCE of 15.79% A5‐Cu reached to 9.3% with a CO_2_ flow rate of 30 sccm (Figure 2f). Additionally, we also compared other commercial AEIs and CEI for CO_2_RR, including Sustainion, Fumion FAA‐3, and Nafion to systematically investigate how ionomer chemistry influences the reaction microenvironment around Cu catalysts based on their distinct main‐side chain structures, ion exchange capacity, and hydrophobic/hydrophilic properties. Their chemical structures were listed in Figure S12. All AEIs modified Cu catalysts showed the enhanced FE and partial current density of C_2+_ products compared to the unmodified Cu catalyst under the optimized current density (Figure S13). Among them, the A5 modified Cu catalyst exhibited the most effective. The FE of C_2+_ products only reached to 43% and 35.7% at 200 mA cm^−2^ for Sustainion and Fumion FAA‐3 modified Cu catalyst, respectively. Moreover, the FE of H_2_ was obviously higher than A‐5 Cu at the setting current density scale, and we also compared the H efficiency and *i‐V* curves among them (Figure S14), the results showed that the ability of *H to participate in the generation of C_2+_ products and active current was significantly inhibited. For Sustainion and Fumion FAA‐3 modified Cu catalyst, the anion conductivity was suppressed and the local microenvironment cannot be stabilized, thus leading to higher H_2_ evolution and lower C_2+_ FE. In addition, the Nafion modified Cu (The structure and morphologydetails can be seen in Figure S15) showed the worst CO_2_RR performance, the FE of H_2_ nearly 100% was detected in the all setting current density, we proposed that this was not merely due to a lack of sufficient anion conduct capacity, but rather to a specific mismatch between the property of Nafion and the reaction microenvironment in the AEM‐MEA system with 0.1 M KHCO_3_ as electrolyte [41]. Compared with the state‐of‐the‐art catalysts in the literature, the 15.79% A5‐Cu performed as one of the best catalysts in the aqueous solution system (Figure 2g and Table S2). In addition, we conducted the durability test in the MEA system at 200 mA cm^−2^, no considerable change was observed about C_2+_ products FE and the applied cell voltage for at least 100 h (Figure 2h). The morphology and structure of 15.79% A5‐Cu catalyst was no obvious difference after long‐time electrolysis confirmed by TEM in Figure S16, and element distribution mappings indicated the ionomer component was not lost.

### 2.3 Effect Factors on CO_2_RR Performance

Second, we started to discuss the effect of reaction condition on CO_2_RR performance. When the KHCO_3_ concentration in the electrolyte was decreased to 0.05 M, the maximum FE and partial current density of C_2+_ products reasonably reduced to 32.7% at 150 mA cm^−2^ for the 15.79% A5‐Cu, and the FE of H_2_ significantly increased owing to the insufficient K^+^ surrounding in the electrolyte; then we raised KHCO_3_ concentration up to 0.2 M, the FE of C_2+_ products slightly decreased to 64.6% at 200 mA cm^−2^; when we further increased KHCO_3_ concentration to 0.5 M, no C_2+_ products was present confirmed by GC and ^1^H NMR, and the FE of H_2_ sharply increased in the all applied current density (Figure S17). It should be noted that only HER process occurred in the 1 M KHCO_3_ aqueous electrolyte at a lower cell voltage. These experimental results concluded a volcano‐shaped trend of C_2+_ products selectivity with KHCO_3_ concentration increased, which was inconsistent with previous majority reports. Based on the above experiment results, we can deduce that K^+^ concentration was not the main factor in regulating the selectivity of C_2+_ products.

To further explain the controversial results of C_2+_ selectivity on KHCO_3_ concentration, we set out to verify the role played by anions and cations. Considering the potential effects of certain specific adsorption anions to interfere experiment results [42, 43], we did not choose the electrolyte such as KCl, K_2_SO_4_ instead by switching the electrolyte from KHCO_3_ to KOH, which holding a higher local pH environment should lead to superior C_2+_ selectivity. However, to our surprise, the C_2+_ products selectivity not yet be improved. In the 1M KOH aqueous electrolyte, almost no CO_2_RR products could be detected on the all tested current density, and the formation rate of H_2_ was faster than 1 M KHCO_3_ due to the highly alkaline environment and lower overpotential of H_2_O dissociation (Figure S18). Meanwhile, we optimized the KOH concentration identical with KHCO_3_ condition, the total C_2+_ products FE (Figure S19) was very similar to the KHCO_3_ system in different concentration and local pH, but the main C_2+_ products switched from EtOH to C_2_H_4_, which can be attributed to local alkaline microenvironment in KOH. According to the results, we can conclude that the anion identity and local pH may not be the main reason for influencing the CO_2_RR performance. We also conducted the durability test in 0.1 M KOH, but the electrolysis operation was failed after about 20 h due to the salt precipitate in the cathode channel (Figure S20).

Moreover, we also considered that the volcano‐shaped relationship of C_2+_ products selectivity may originate from the potential dependence in the constant current electrolysis mode. According to previous reports, there is usually an optimal potential window within which C_2+_ selectivity is highest, excessive positive or negative can lead to a decrease in selectivity [44, 45], we made the applied cell voltage negative shift from −2.68 V (200 mA cm^−2^, 1 M KHCO_3_) to −3.23 V (200 mA cm^−2^, 0.1 M KHCO_3_) in constant potential electrolysis mode. The constant potential electrolysis results showed that there was still no C_2+_ products presence in 1 M KHCO_3_ electrolyte. Based on the results, the potential‐selectivity dependence relationship induced by concentration differences can also be ruled out.

### 2.4 The Revelation of the Confined Effect by Ionomer Addition

**FIGURE 3:**
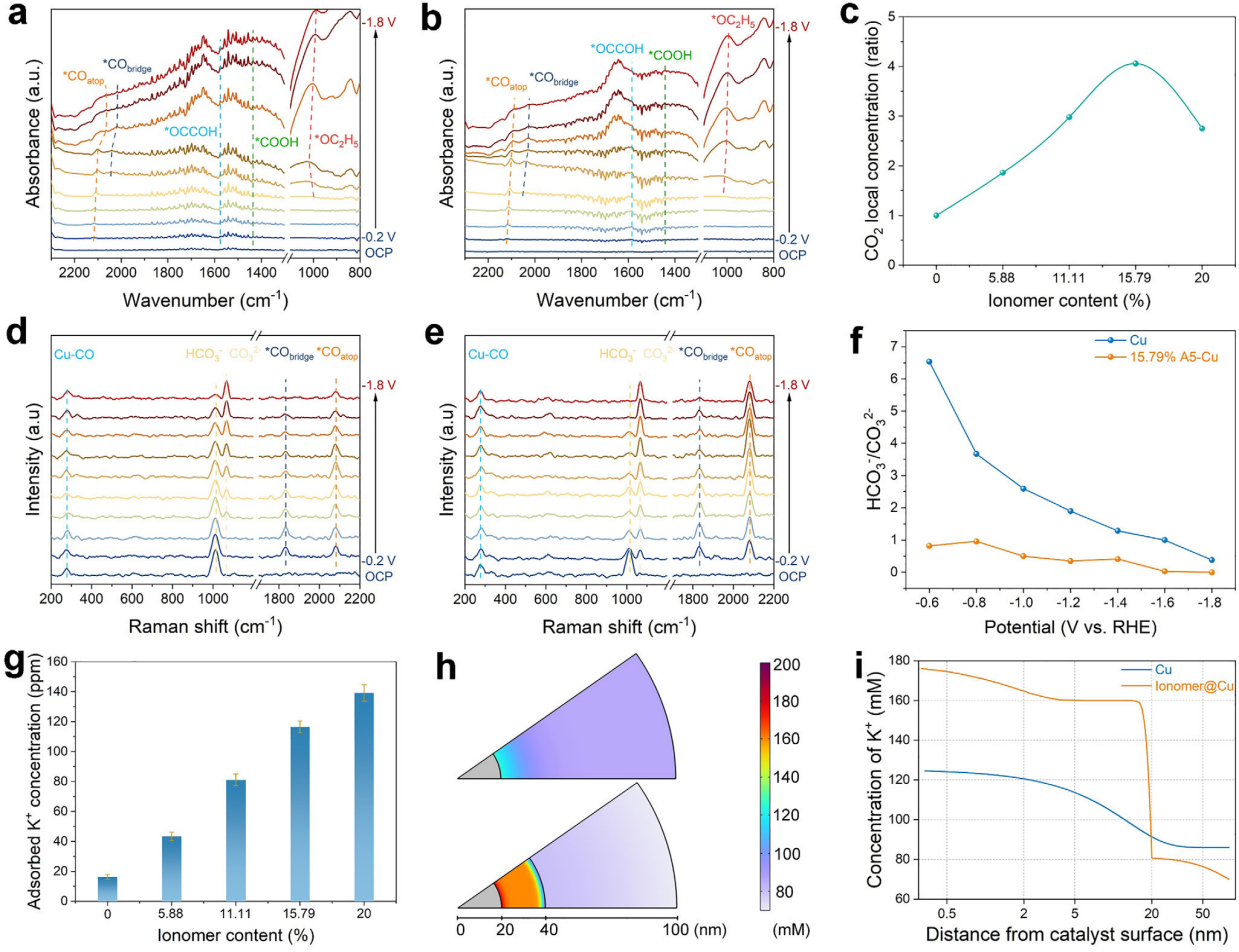
(a) In situ Raman spectra of Cu catalyst. (b) In situ Raman spectra of 15.79% A5‐Cu catalyst. (c) The relative CO_2_ local concentration of different ionomer content Cu catalysts determined by the electrochemical probe method. (d) In situ ATR‐SEIRAS spectra of Cu catalyst. (e) In situ ATR‐SEIRAS spectra of 15.79% A5‐Cu catalyst. (f) The value of local HCO_3_^−^/CO_3_^2−^ over Cu and ionomer @Cu catalysts at varying applied potentials. (g) Adsorbed K^+^ concentration of different ionomer content Cu catalysts determined by ICP‐OES. (h) Finite element method simulation concentration contour plot of K^+^ over Cu and ionomer@Cu catalysts. (i) Finite element method simulation concentration curves of K^+^ over Cu and ionomer@Cu catalysts.

In situ ATR‐SEIRAS and Raman spectroscopy (Figure S21) was applied to disclose the interface microenvironment change after ionomer introduction. As shown in Figure 3a,b, peak at ∼1440 cm^−1^ was attributed to *COOH, a critical precursor for *CO formation. In addition, the peak appeared at ∼1578 and ∼1017 cm^−1^ was attributed to *OCCOH and *OC_2_H_5_, which was critical intermediate for C_2_H_4_ and CH_3_CH_2_OH generation via C─C coupling, respectively. In the *CO intermediate region, there were two peaks locate at ∼2102 and ∼2040 cm^−1^ for the bare Cu and 15.79% A5‐Cu catalyst, which are associated with the atop‐bound *CO (*CO_atop_) and bridge‐bound *CO (*CO_bridge_), respectively [46, 47, 48]. To be noted, the both *CO intermediate signal intensity for 15.79% A5‐Cu was significantly stronger than the bare Cu over the potential range, and the *CO_bridge_ intermediate exist in the applied potential range from −0.8 to −1.8 V on 15.79% A5‐Cu catalyst, but only appear from −1.0 to −1.8 V on Cu, these evidences indicate that 15.79% A5‐Cu catalyst owns a higher *CO coverage, which facilitate the generation of C_2+_ products. The *CO enrichment ability of the catalysts was also confirmed by the electrochemical probe method using 1,4‐benzoquinone as probe molecule since the CO_2_ local concentration is the decisive factor to influence the *CO coverage [49, 50]. As shown in Figure S22 and Table S3 and Figure 3c, compared with the bare Cu catalyst, the local CO_2_ concentration reach the maximum at 15.79% ionomer addition content, further increasing the ionomer addition content to 20% would lead to a decrease. These experimental results implied that although an appropriate A5 addition content can boost the CO_2_ diffusion to the Cu surface and ion transport in the reaction interface, but excessive ionomer may block the active site which used for CO_2_ activation and conversion on Cu surface.

In the in situ Raman spectroscopy for the bare Cu and the 15.79% A5‐Cu catalyst, as shown in Figure 3d,e, no obvious signals assigned to Cu^+^ were detected in the ∼520 and ∼621 cm^−1^ region, indicating the Cu^2+^ was fully reduced to Cu^0^, which is consistent with the result of XRD and XPS. The peak located at ∼278 cm^−1^ was observed on both bare Cu and 15.79% A5‐Cu catalysts, corresponding to the restricted rotation of the *CO stretching mode [51]. Furthermore, the peaks for adsorbed *CO intermediate in the form of bridge‐bound *CO_bridge_ (located at ∼1833 cm^−1^) and atop‐bound *CO_atop_ (located at ∼2082 cm^−1^) were also detected [52, 53]. As the applied potential gradually shifted negative, the signal intensity of these two *CO stretching mode first increased and then decreased, meanwhile the signal intensity of *CO_bridge_ and *CO_atop_ for 15.79% A5‐Cu catalyst was significantly higher than the bare Cu. The Raman results showed that the introduction of ionomer indeed increased the local CO_2_ concentration, which consistent with the ATR‐SEIRAS and electrochemical probe experiment. Peaks located at ∼1013 and ∼1065 cm^−1^ were ascribed to the adsorbed HCO_3_^−^ and CO_3_^2^^−^ on Cu and 15.79% A5‐Cu catalyst surface, respectively. Generally, the local pH can be evaluated by monitoring the ratio of HCO_3_^−^/CO_3_^2^^−^ during CO_2_RR, where the lower ratio value of HCO_3_^−^/CO_3_^2^^−^ means the higher local pH around catalyst [54, 55]. As shown in Figure 3f, we can see that in the applied potential range from −0.6 to −1.8 V, the quantitative ratio (Figure S23 and Table S4) of HCO_3_^−^/CO_3_^2^^−^ for 15.79% A5‐Cu catalyst was significantly lower than bare Cu, this demonstrated the introduction of ionomer could create a higher local pH microenvironment, which was beneficial for C─C coupling.

To further reveal the role of ionomer after introduced on Cu, K^+^ retention experiment was conducted to investigate the adsorbed K^+^ concentration in the confined space [56, 57]. As shown in Figure 3g and Figure S24, the adsorbed K^+^ concentration maintained continuous rise with ionomer addition content, and the K^+^ concentration of ionomer‐modified Cu was higher than the bare Cu without ionomer. In common, the free H_2_O (K^+^‐H_2_O) proportion increases when K^+^ concentration increase as K^+^ was used to polarize H_2_O molecules and thereby lower the H_2_O dissociation energy. To detect local K^+^ and OH^−^ concentration distribution after ionomer introduction, the finite element simulations were employed with a sector model as shown in Figure 3h,i, the simulations indicated that compared with the bare Cu, the local K^+^ concentration was obviously higher than the bulk concentration within a range from catalyst surface to near 20 nm, once the ionomer thickness beyond 20 nm, K^+^ concentration dropped sharply due to the diffusion restriction caused by ionomer. For the OH^−^ curves, the local OH^−^ concentration was enriched compared with the bare Cu after ionomer introduction within the whole simulation thickness scale (Figure S25). Unlike the K^+^ enrichment effect of CEIs, this phenomenon of K^+^ concentration higher than the bulk electrolyte may be attributed to that the cationic‐anionic network of the confined ionomer layer acted as a reservoir for K^+^, facilitating the enrichment of K^+^ near the active sites [58]. These results suggested ionomer would induce enrichment of OH^−^ and confinement of K^+^. This higher local pH microenvironment was consistent with the in situ Raman results, which could facilitate C_2+_ products generation.

### 2.5 Interfacial H_2_O Configuration Characterizations and Mechanism Discussion

**FIGURE 4:**
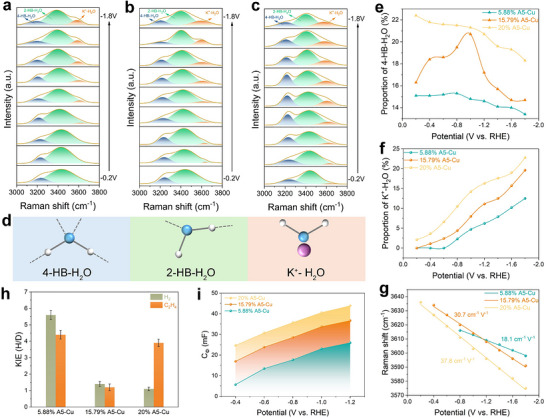
In situ Raman spectra of interfacial water in the region of 3000–3800 cm^−1^ at varying applied potentials during CO_2_RR for (a) 5.88% A5‐Cu catalyst (b) 15.79% A5‐Cu catalyst. (c) 20% A5‐Cu catalyst. (d) The chemical structure of 4‐HB‐H_2_O, 2‐HB‐H_2_O, and K^+^‐H_2_O. (e) The proportion of 4‐HB‐H_2_O over 5.88%, 15.79% and 20% A5‐Cu catalysts at varying applied potentials during CO_2_RR. (f) The proportion of K^+^‐H_2_O over 5.88%, 15.79%, and 20% A5‐Cu catalysts at varying applied potentials during CO_2_RR. (g) The frequency plot of changes in the O−H stretching modes in Raman spectra of interfacial water. (h) The KIE value of H_2_ and C_2_H_4_ over 5.88%, 15.79%, and 20% A5‐Cu catalysts. (i) The calculated C_Φ_ value at varying applied potentials based on EIS fitting over 5.88%, 15.79% and 20% A5‐Cu catalysts in 0.1 M KHCO_3_.

According to previous reports, confined H_2_O in distinct local microenvironment can display significantly different behaviors from that bulk H_2_O. The K^+^‐H_2_O configuration, which has the strongest ability to release *H, is the direct source of *H. In addition, the robust hydrogen bond network in the 4‐HB‐H_2_O configuration could enhance proton transfer via the Grotthuss mechanism to promote hydrogenation of *CO intermediate. To confirm the confined H_2_O introduced by A5 ionomer affects the selectivity of C_2+_ products, the in situ Raman spectroscopy was applied to track the interfacial H_2_O configuration evolution with the potential in the range of 3000–3800 cm^−1^ (Figure 4a–c). Through Gaussian fitting, the O─H stretching model can be deconvoluted into three compoents: 4‐coordinated hydrogen‐bond H_2_O (4‐HB‐H_2_O), 2‐coordinated hydrogen‐bond H_2_O (2‐HB‐H_2_O), and cation‐hydrated H_2_O (K^+^‐H_2_O), corresponding to ∼3200, ∼3400, and ∼3600 cm^−1^, respectively [59, 60] (Their structure models were shown in Figure 4d). The proportion of these three H_2_O configurations with applied potential was depicted in Figure 4e,f and Figure S26. As the applied potential gradually shifted negative, for the 5.88% A5‐Cu, the proportion of 4‐HB‐H_2_O maintain relatively stable in the potential range from −0.2 to −1.8 V. For the 15.79% A5‐Cu, the proportion of 4‐HB‐H_2_O was first increased and then decreased, reaching to 20.7% as the peak at −1.0 V. For the 20% A5‐Cu, although the proportion of 4‐HB‐H_2_O was also gradually decreased, but its proportion was signficantly higher than 5.88% and 15.79% A5‐Cu. Considering the proportion of 2‐HB‐H_2_O, its proportion both gradually decreased in the three Cu catalysts; in contrast, for the both three Cu catalysts, the proportion of K^+^‐H_2_O gradually increased in the whole potential range, from 0 to 12.5%, 0 to 19.6%, and 2.1% to 22.8%, respectively. It is worth noting that the 20% A5‐Cu catalyst held the highest proportion of K^+^‐H_2_O at each potential. However, the proportion of 2‐HB‐H_2_O for 5.88% A5‐Cu ranked the highest among the three Cu catalysts, these opposite results indicated that high concentrations K^+^ might disrupt the original hydrogen bonding network of 2‐HB‐H_2_O, inducing 2‐HB‐H_2_O re‐coordinate to form K^+^‐H_2_O configuration. The variation of the vibration frequency of H_2_O as a function of applied potential has been attributed to the Stark effect, the slope magnitude of vibration frequency‐applied potential curve means the sensitivity about the H_2_O with applied potential. As shown in Figure 4g, the Stark tuning rate of K^+^‐H_2_O configuration showed in the order of 20% A5‐Cu (37.8 cm^−1^ V^−1^), 15.59% A5‐Cu (30.7 cm^−1^ V^−1^), and 5.88% A5‐Cu (18.1 cm^−1^ V^−1^), which was similar with the trend of K^+^‐H_2_O configuration proportion. Obviously, the proportion of interfacial H_2_O configurations dynamically varies with its confined microenvironment. K^+^‐H_2_O configuration dominates the *H generation process by promoting H_2_O dissociation, and 4‐HB‐H_2_O confirguration dominates the *H transfer process through the robust hydrogen bond network. Meanwhile, 2‐HB‐H_2_O confirguration serves as the bridge connecting the other two configurations, maintaining a dynamic and controllable interfacial microenvironment. The synergistic effect among the three interfacial H_2_O configurations enables the precise release and efficient transfer of *H species, boosting the CO_2_RR performance.

As mentioned above, the H source of CO_2_RR products is H_2_O, the K^+^‐H_2_O configuration has the strongest *H release ability, and the released *H could fastly transfer to the *CO intermediate through the robust hydrogen bond work of 4‐HB‐H_2_O, so accelerating the ability of *H generation and *H transfer, improving the *H utilization efficiency, helps to improve the selectivity of C_2+_ products. In other words, we can predict that if the *H generation was limited, the HER activity and *CO hydrogenation and subsequent C─C coupling all would be suppressed. If the *H transfer was limited, the *H species was unable to promptly transfer to *CO intermediate, the excessive *H accumulation would enhance HER performance. To confirm the effect of *H generation ability on CO_2_RR performance, we replaced 0.1 M KHCO_3_ with [K^+^] = 0.1 M KH_2_PO_4_/K_2_HPO_4_ buffer solution as electrolyte due to stronger *H supplied ability and pH buffer capacity. The experiment results showed that compared with 0.1 M KHCO_3_, for the 5.88% A5‐Cu catalyst, the FE of H_2_ and C_2+_ products was promoted compared with 0.1 M KHCO_3_ under optimized condition, and the total current response was also enhanced seen from the LSV curves (Figure S27). After switching to KH_2_PO_4_/K_2_HPO_4_ buffer solution, there were almost no significant impact on FE of H_2_ and C_2+_ products and also total current response for 15.79% A5‐Cu (Figure S28). However, for the 20% A5‐Cu, the FE of H_2_ was obviously increased but the selectivity of C_2+_ products markedly decreased, and the decreased total current response was also seen (Figure S29). These results suggested *H generation was limited in 5.88% A5‐Cu, rather than in 15.79% and 20% A5‐Cu.

Moreover, the kinetic isotope effect (KIE) was measured to evaluate the *H generation and transfer kinetics. If *H generation (H_2_O dissociation) is involved in the rate‐determining step (RDS), the KIE value is 2–7, which called as the KIE [61, 62]; if *H transfer (*H transfer to *CO) is involved in the RDS, the KIE value is usually determined by the type of product. For the 5.88% A5‐Cu catalyst, the KIE values of H_2_ and C_2_H_4_ were 5.6, 4.4, respectively. This indicated that *H indeed originates from H_2_O dissociation and the sluggish H_2_O dissociation limits the CO_2_RR kinetics over 5.88% A5‐Cu. In addition, the KIE values of H_2_ and C_2_H_4_ were 1.4 and 1.2 for 15.79% A5‐Cu, but were 1.1 and 3.9 for 20% A5‐Cu. The gradually decreasing KIE of H_2_ indicated that as A5 ionomer content increases, the ability of *H generation gradually strengthens, and the KIE of H_2_ and C_2_H_4_ all closed to 1 showed the *H generation and transfer may reach balance for 15.79% A5‐Cu (Figure 4h). It should be noted that the C_2_H_4_ formation requires *H transfer and C─C coupling process, this mismatch between supply and demand of *H species can lead to significant KIE effects for C_2_H_4_, the KIE of C_2_H_4_ in three catalyst models implied that the RDS for C_2+_ products was *H transfer step rather than C─C coupling. To investigate the *H coverage during the H_2_O dissociation process on the catalyst surface, in situ electrochemical impedance spectroscopy (EIS) was conducted [63]. The double‐parallel equivalent circuit model (Figure S30) was used to simulate the Nyquist plots and the fitting plots were shown in Figure S31. The adsorption pseudo‐capacitance (C_φ_) referring the *H adsorption behavior can be reflected the *H coverage on catalyst surface. As illustrated in Figure 4i, the higher C_φ_ value at every same applied potential can be seen with the increased A5 ionomer content. Obviously, the *H coverage on catalyst surface was promoted with the ionomer addition. Therefore, combining the KIE and in situ EIS and Raman results, we concluded the confinement of ionomer restructured the configuration proportion of interfacial H_2_O, promoting the H_2_O dissociation, but only a balanced relationship between *H generation and *H transfer could most boost the C_2+_ products generation.

### 2.6 Theoretical Calculation

**FIGURE 5:**
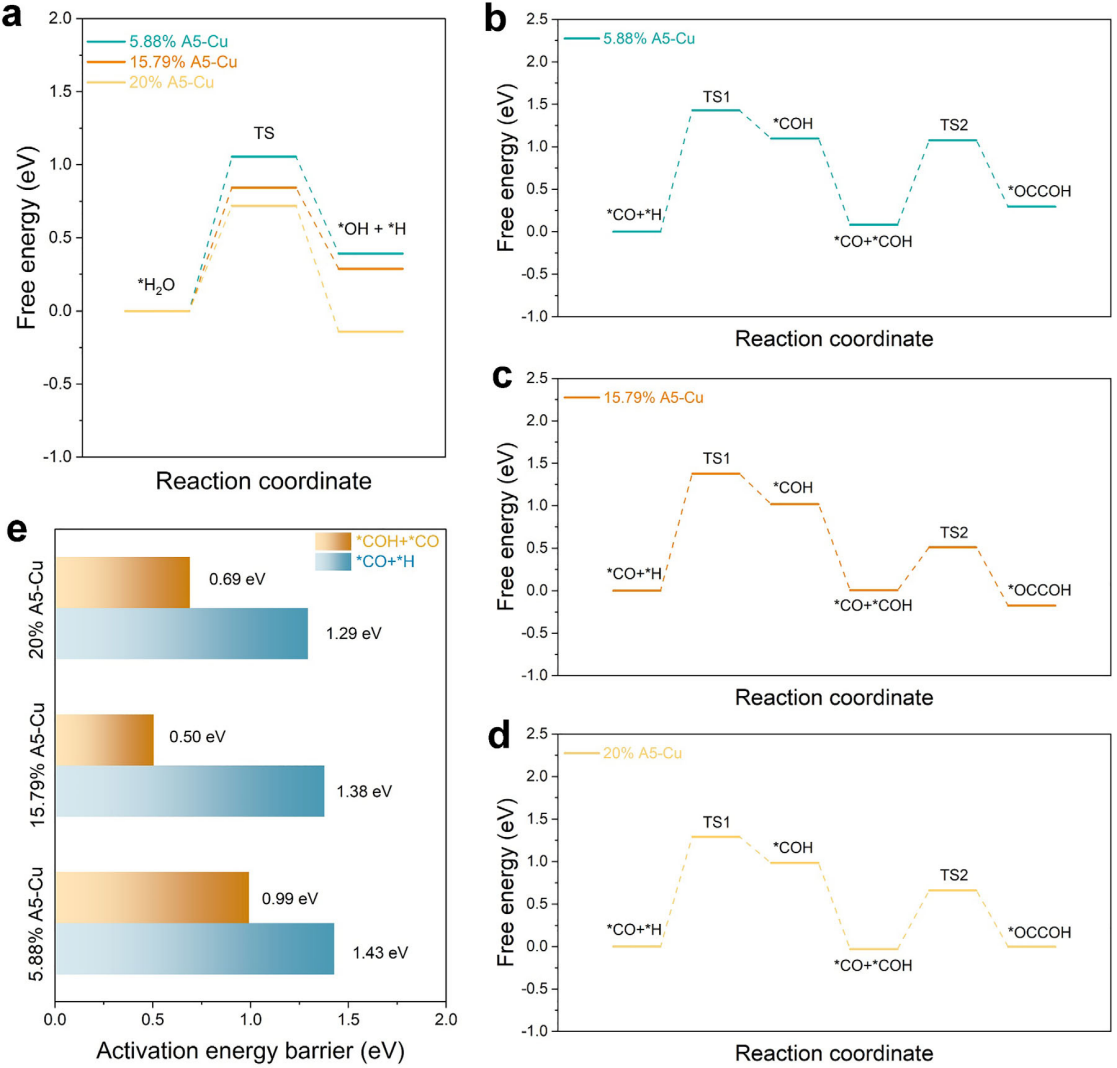
(a) The H_2_O dissociation energy barrier on Cu(111) facet over 5.88%, 15.79%, and 20% A5‐Cu catalyst models, respectively. The free energy diagram of *CO converted to *OCCOH intermediate in C_2+_ products generation steps for (b) 5.88% A5‐Cu catalyst. (c) 15.79% A5‐Cu catalyst. (d) 20% A5‐Cu catalyst. (e) The comparison of activation energy barrier between *CO+*H and *CO+*COH step over 5.88% A5‐Cu, 15.79% and 20% A5‐Cu catalysts, respectively.

DFT calculations were conducted to further investigate the *H species microenvironment effect on products selectivity. Based on the results of experiment characterization, we calculated the energy barrier of reaction pathways for H_2_O dissociation and *OCCOH intermediate formation from *CO on Cu (111) facet in different *H coverage. All the optimized adsorption models and detailed data were shown in Figures S32–S37. As shown in Figure 5a, the formation of the transition state (TS) from *H_2_O displayed the highest energy barrier which acted as the rate‐determining step for H_2_O dissociation [64]. With the increased K^+^‐H_2_O proportion, the energy barrier of H_2_O dissociation was gradually decreased. To be more detailed, the energy barrier of H_2_O dissociation was 1.06, 0.84, and 0.72 eV for 5.88%, 15.79%, and 20% A5‐Cu catalyst, respectively. Moreover, the *Δ*Gibbs free energy (*Δ*G) shifts from 0.39 to −0.14 eV (Figure S38). These results clearly demonstrated that the K^+^‐H_2_O configuration of interfacial H_2_O could significantly promote the H_2_O dissociation at the interfacial sites, thereby accelerating the subsequent hydrogenation process.

Based on the results of EIS fitting, we selected three *H coverage models (2/16, 4/16, and 8/16 ML for 5.88%, 15.79%, and 20% A5‐Cu catalyst, respectively) to simulate the effect on the reaction pathways from *CO to *OCCOH, which was generally considered as the critical intermediate for C_2+_ products generation. As shown in Figure 5b–d, the released *H was first transfer to *CO intermediate to form *COH through hydrogen‐bond network of 4‐HB‐H_2_O, and then the *COH was further coupled with *CO to form *OCCOH. We can find that the energy barrier of *COH formation is significantly higher than *OCCOH formation in both three calculation models, this result indicated that the rate‐determining step for C_2+_ products formation was the *CO hydrogenated to *COH step rather than the asymmetric *COH‐*CO coupling step [65, 66]. The calculation results were consistent with the KIE experiment. In addition, with the increasing *H coverage, the activation energy barrier of *CO hydrogenation gradually decreased from 1.43 to 1.29 eV, but the activation energy of *CO coupling with *COH was first decreased and then increased, reaching the minimum value 0.50 eV at 4/16 ML (Figure 5e), meanwhile the C─C coupling step was the most energetically favorable (Figure S39). The calculation results showed that although the enhanced *H coverage can promote the *H transfer to *CO to form *COH, but only a moderate *H availability could accelerate *COH‐*CO coupling. If the *H coverage further increases, the HER performance would dominate and suppress the C_2+_ products selectivity.

## 3 Conclusion

In summary, we elucidated the crucial role of interfacial water configuration in steering the selectivity toward C_2+_ products during CO_2_ electroreduction by ionomer‐confined Cu catalyst. In situ spectroscopy revealed that the confinement effect significantly modulates the proportion of K^+^‐H_2_O and 4‐HB‐H_2_O at the interface. The K^+^‐H_2_O can facilitate H_2_O dissociation, enhancing its proportion improves the *H supply; increasing the proportion of 4‐HB‐H_2_O, which functions as an efficient *H transfer network, can accelerate the migration of *H to adsorbed *CO intermediates. DFT calculations further demonstrated that a moderate *H availability induced by the balanced proportion of K^+^‐H_2_O and 4‐HB‐H_2_O configurations effectively lowers the energy barrier for *CO hydrogenation to *COH and promotes *H utilization efficiency. Importantly, higher *CO coverage, which is maintained under the confined microenvironment, is identified as another non‐negligible factor contributing to the improved C_2+_ products selectivity. By optimizing the ionomer content, the confined catalyst achieves a high Faradaic efficiency of 82% for C_2+_ products at 200 mA cm^−2^ in 0.1 M KHCO_3_, maintaining stable operation for over 100 h without salt precipitation. This work underscores that beyond *H generation, the H transfer process also plays an equally critical role in determining the selectivity of C_2+_ products in CO_2_RR systems.

## Author Contributions

**Dawei Zhou**: investigation, methodology, data curation, validation, writing – original draft. **Songhu Bi**: investigation, validation. **Jie Zhang**: validation. **Jinghui Deng**: validation. Keyi Xu: visualization. Lin Xia: funding acquisition, supervision, visualization.

## Conflicts of Interest

The authors declare no conflicts of interest.

## Supporting information




**Supporting File**: advs76470‐sup‐0001‐SuppMat.docx. The supporting File should choose the "SI‐Revised Tracked changes" we upload latestly

## Data Availability

The data that support the findings of this study are available in the supplementary material of this article.
