# Supplementary material for: Promoting Electroreduction CO2 to Multi‐Carbon Products by Tailoring the *H Availability With Optimized Interfacial H2O Configuration
Source: Adv Sci (Weinh). 2026 Jul 17;13(54):e76470. doi: 10.1002/advs.76470 (PMC13379214; doi:10.1002/advs.76470)
Supplement: Supplementary file 1 [file ADVS-13-e76470-s001.docx]

**Promoting Electroreduction CO_2_ to Multi-carbon Products by Tailoring the *H Availability with Optimized interfacial H_2_O Configuration**

Dawei Zhou* ^[a]^, Songhu Bi ^[a]^, Jie Zhang ^[a]^, Jinghui Deng ^[a]^, Keyi Xu ^[b]^, Lin Xia* ^[a]^

[a]	D. Zhou, S. Bi, J. Zhang, J. Deng, L. Xia
Key Laboratory of Quantitative Synthetic Biology, Shenzhen Institute of Synthetic Biology, Shenzhen Institute of Advance Technology, Chinese Academy of Science,

Shenzhen 518055, China

E-mail: dw.zhou@siat.ac.cn; lin.xia@siat.ac.cn

[b]	K. Xu

Shenzhen Powered carbon Biotechnology Co., Ltd.,

Shenzhen 518055, China

**Experimental Section**

**Chemicals and Materials.** Copper (II) nitrate trihydrate (Cu(NO_3_)_2_·3H_2_O, 99%), 1,4-benzoquinone, potassium bicarbonate (KHCO_3_, 99.5%), sodium hydroxide (NaOH, ≥95%), potassium hydroxide (KOH, ≥95%), tetrabutylammonium hexafluorophosphate ([Bu_4_N][PF_6_], ≥98%), KH_2_PO_4_/K_2_HPO_4_ buffer solution were purchased from Shanghai Macklin Biochemical Co., Ltd. Methanol (MeOH, ≥99.7%), ethanol (EtOH, ≥99.7%), isopropanol (*i*-PrOH, ≥99.7%), ammonia aqueous solution (NH_3_·H_2_O, 25~28 wt%), dimethylformamide (DMF, ≥99%) were obtained from Sinopharm Chemical Reagent Co., Ltd. IrO_2_@Ti felt was purchased from Hefei Momenta Energy CO., Ltd. Anion-exchange membrane (Versogen, PiperION-A40-HCO_3_^-^, 40 μm), anion-exchange ionomer (AEI, PiperION-A5), polytetrafluoroethylene-hydrophobized carbon paper (PTFE-CP, YLS-30T GDL), Nafion D-521 dispersion (5% w/w in water and 1-propanol, ≥0.92 meg/g exchange capacity), Sustainion XA-9 (5% w/w in ethanol), Fumion FAA-3 (10% w/w in DMF) were purchased from Suzhou Sinero Technology Co., Ltd.

**Preparation of the precursor nano CuO.**

The nano CuO were fabricated through the annealing of Cu(OH)_2_ precursor. Typically, 20 g Cu(NO_3_)_2_·3H_2_O was dissolved in 2 L deionized water and stirred for 30 min to form blue solution, and then 600 mL ammonia aqueous solution (0.15 mol L^-1^) was added to the Cu(NO_3_)_2_ solution with a speed of 10 mL min^-1^ under constant stirring at room temperature. A blue precipitate of Cu(OH)_2_ was obtained when dropwise added 200 mL of NaOH (1 mol L^-1^) solution (10 mL min^-1^) to the above solution. After this process was finished, the blue Cu(OH)_2_ precipitate was filtered washed for several times with deionized water and then dried in a freeze dryer overnight. Finally, the nano CuO was prepared by annealing the Cu(OH)_2_ in the air atmosphere at 500 ℃ for 2 h with a heating rate of 5 ℃ min^-1^.

**Preparation of Cu and anion-exchange ionomer modification Cu catalysts.**

For the 15.79% A5-Cu as an example. Generally, 720 mg of as-prepared nano CuO was dispersed in 103.2 mL isopropanol to ultrasonic in a cell pulverization machine for 30 min under the ice-bath condition, then 1080 mg PiperION-A5 diluted solution (10 wt% dispersed in ethanol) was added into the above solution for another 30 min ultrasonic under the ice-bath condition. Next, the as-prepared catalyst ink was sprayed on the YLS-30T GDL uniformly by a spray machine to keep the ~0.5 mg cm^-2^ catalyst loading, the obtained 13.04% A5-CuO GDL electrode was dried for the subsequent electrochemical testing experiments. Finally, an electrochemical activated step was carried out for 1h through *in-situ* electroreduction in the MEA at 100 mA cm^-2^ under CO_2_ atmosphere in 0.1 M KHCO_3_, the 15.79% A5-Cu catalyst was obtained until the activated process was finished. The preparation methods of Cu, 5.88%, 11.11% and 20% A5-Cu catalysts, modified without or with different A5 addition ratio, were similar with the above steps. The corresponding addition amounts of ionomer and volumes of isopropanol were listed in the Table S1.

**Preparation of others ionomer modification Cu catalysts.**

The preparation method of others ionomer modification Cu catalyst was similar with the A5 modification Cu catalysts expect that the added ionomer was Nafion, Sustainion XA-9, Fumion FAA-3 dispersion, respectively. The corresponding parameters were listed in the Table S1.

**Characterization and measurements.**

The morphology of catalysts was characterized by a Thermofisher Apero2S HiVac scanning electron microscope (SEM) at 5 kV and transmission electron microscope (TEM, JEM-F200) equipped with energy dispersive X-ray spectroscopy (EDS) operated at 200 kV. Powder X-ray diffraction (XRD) patterns were acquired with a X-ray diffractometer (Bruker, D8 Advance) with Cu-Kα radiation, and the scan speed was 20 ℃/min. X-ray photoelectron spectroscopy (XPS) study was conducted on a Supra surface analysis instrument (Thermo scientific, Escalab xi^+^) using a monochromatic Al Kα X-ray beam (1486.6 eV). The contaminated carbon C 1s signal at 284.8 eV was used to calibrate binding energies. The stage was evacuated in vacuum to prevent the samples to be oxidized in the air.

**Electrocatalytic CO_2_ reduction**

Electrochemical tests were conducted in a typical zero gap MEA cell (work area 2.5 $\times$ 2.5 cm^2^) which included a gas diffusion channel, a cathodic compartment and an anodic compartment. An anion exchange membrane (PiperION-A40-HCO_3_^-^) was used to separate the anodic and cathodic chambers, and IrO_2_@Ti was used as the counter electrodes. The electrolysis was conducted using a DC power supply device. In a typical electrochemical test, 0.1 M KHCO_3_ was used as the electrolyte, and it was circulated through the anodic chambers using peristatic pumps at a rate of 30 mL min^-1^. Unless stated otherwise, the flow rate of CO_2_ gas through the gas channel was controlled to be 30 sccm using a digital gas flow controller.

**Gaseous and liquid products analysis**

The gaseous products were collected and quantified by an on-line gas chromatography (GC) (GC 8890) system, equipped with thermal conductivity detector (TCD) using Argon as the carrier gas. The liquid product was analyzed by 1H NMR (Bruker Advance III 500 HD spectrometer) in D_2_O, employing H_2_O suppression mode. Dimethyl sulfoxide (DMSO) was the reference for ethanol, acetic acid and n-propanol, and the phenol was the reference for formate. The Faradaic efficiency (FE) of products was calculated by the equation:

$$FE= \frac{znF}{Q} \times100\%$$

Where z represents the number of electrons transferred for every specific product formation, n is the mole of product obtained from GC or ^1^H NMR, F is Faraday constant (96485 C/mol) and the Q is the amount of electrolysis charge recorded by the DC power supply device.

SPCE for C_2+_ products were calculated based on the following equation:

$SPCE= \frac{60s \times\sum(I \times xi \times FEi \div\left( z \times F \right))}{V \left( L/min \right)\times1\min\div24.5 (L/mol)}$

Where *I* is the applied total current, FE_i_ is the FE of a specific product from CO_2_ reduction, x_i_ is the mole ratio of CO_2_ to a specific product (for example, x_i_ = 1 for C_1_ products while x_i_ = 2 for C_2_ products) and z is the number of electron of transfers for every specific product molecule, V is the CO_2_ flow rate.

The *H utilization efficiency for HER and for C_2+_ products generation were calculated by these following equations:

*$H_{HER}= \frac{FE\left( H2 \right)\times2}{2} \times100\%$

***$H_{C2+}= \frac{FE\left( C2H4 \right) \times\frac{4}{12} +FE\left( EtOH \right)\times\frac{6}{12} +FE\left( CH3COOH \right) \times\frac{4}{8} + FE\left( PrOH \right)\times\frac{8}{18}}{FE\left( H2 \right) \times\frac{2}{2} +FE\left( CH4 \right) \times\frac{4}{8} +FE\left( HCOOH \right) \times\frac{2}{2} + FE\left( C2H4 \right) \times\frac{4}{12} +FE\left( EtOH \right)\times\frac{6}{12} +FE\left( CH3COOH \right) \times\frac{4}{8} + FE\left( PrOH \right)\times\frac{8}{18}}$

**In situ Raman measurements**

Raman spectra were collected using a commercially available Raman cell. For in situ measurements, Raman spectra were acquired using a 532 nm excitation laser and an 800 grooves/mm diffraction grating (Renishaw, inVia Reflex). The laser power was maintained at 5 mW. During the measurement, the pump was employed to circulate the electrolyte at a constant flow rate of 30 mL min^-1^, meanwhile the CO_2_ flow was continuous supplied by a mass flow controller. The graphite electrodes and Ag/AgCl (in saturated KCl solution) were utilized as the counter and reference electrode respectively, the catalyst was dropped on the GDL uniformly. In all tests, the CO_2_-saturated 0.1 M KHCO_3_ aqueous solution served as the catholyte and anolyte. Before measurements, the working electrode was activated lasting for a time until the system was stabilized. Subsequently, the signals were recorded from OCP and scan from -0.2 to -1.8 V (vs. RHE) with a 0.2 V step. The digital photo was shown in Figure S18a.

**In situ ATR-FTIRS measurements**

A commercially available spectro-electrochemical cell equipped with three electrodes, including a Si prism coated with a 100 nm Au film as the working electrode, a platinum-wire as the counter electrode, and a standard Ag/AgCl (in saturated KCl solution) electrode as the reference, was utilized for ATR-SEIRAS measurements. The catalyst suspension was dropped onto the prepared silicon/Au prism. All electrochemical tests were conducted in a CO_2_-saturated 0.1 M KHCO_3_ aqueous solution under a continuous CO_2_ flow. Before conducting the electrochemical measurements, the working electrode was activated at 5 mA cm^-2^ until the system was stabilized. Subsequently, the signals were recorded from OCP and scan from -0.2 to -1.8 V (vs. RHE) with a 0.2 V step. All ATR-SEIRAS spectra were recorded by a Fourier Transform Infrared Spectrophotometer (FT-IR, Nicolet iS50) equipped with a mercury cadmium telluride detector. The digital photo was shown in Figure S18b.

**Finite-element method (****FEM) simulations**

The modeling of the ions accumulation-diffusion process was performed in COMSOL Multiphysics software with diluted species transport module. This simulation models the variation of K^+^ concentration with distance x on the surface of a Cu electrode in a bulk solution of 0.1 M KHCO₃. Different thicknesses of the ionomer coating (confined space) on the Cu electrode surface leaded to distortions in the K^+^ concentration distribution. Two distinct models were constructed in the simulation:

1. Control sample: Cu electrode (20 nm thick) + electrolyte region (80 nm thick);
2. Experimental sample: Cu electrode (20 nm thick) + ionomer (20 nm) + electrolyte region (60 nm thick).
3. The initial material concentration in both cases is 0.1 M (100 mol/m³).

The structure of the simulation model is illustrated in the figure below:

The following is the theoretical methodology section.

Based on the steady-state continuity equations and the law of mass conservation, we have:

$$\frac{\partial ci}{\partial t}+ \nabla\cdot Ni=0$$

The boundary condition was set as followed:

$$\vec{n}\cdot J=0$$

where J means the species flux on the boundary face.

The control equations for electrochemical reactions and concentration distribution are as follows：

$$\frac{\partial ci}{\partial t}+ \nabla\cdot Ji+u \cdot\nabla ci=Ri$$

It can be seen from electrochemical reactions act as source terms, providing reactants to the diffusion equation. The reaction rate follows the Arrhenius reaction formula:

$$R =\mathrm{Ae}^{-E_{a}/RT}$$

In the equation, c is the concentration, J represents the flux of diffusion, R is the reaction rate, k is the rate constant; A is the characteristic constant of the given reaction, known as the pre-exponential factor; e is the base of natural logarithm (2.718); R is the gas constant (8.314 J mol^-1^ K^-1^), Ea is the activation energy of the reaction.

**DFT calculations method**

We have employed the first-principles ^[1,2]^ to perform all density functional theory (DFT) calculations within the generalized gradient approximation (GGA) using the Perdew-Burke-Ernzerhof (PBE) ^[3]^ formulation. We have chosen the projected augmented wave (PAW) potentials ^[4,5]^ to describe the ionic cores and take valence electrons into account using a plane wave basis set with a kinetic energy cutoff of 450 eV. Partial occupancies of the Kohn−Sham orbitals were allowed using the Gaussian smearing method with a width of 0.05 eV. The electronic energy was considered self-consistent when the energy change was smaller than 10^−6^ eV. A geometry optimization was considered convergent when the energy change was smaller than 0.02 eV Å^−1^. The vacuum spacing in a direction perpendicular to the plane of the structure is 20 Å for the surfaces. The Brillouin zone integration is performed using 2×2×1 Monkhorst-Pack k-point sampling for a structure. The climbing image-nudge elastic band (CI-NEB) method was used to calculate the energy barrier of reaction on the substrates.

Figure S1: The XRD pattern of nano CuO and different ionomer content modify A5-CuO catalyst precursors.

Figure S2: SEM images of different catalyst precursors. (a) nano CuO; (b) 4.76% A5-CuO; (b) 9.09% A5-CuO; (d) 13.04% A5-CuO; (e) 16.67% A5-CuO; (f) 13.04% Nafion-CuO.

Figure S3: nano CuO precursor. (a) TEM image; (b) HR-TEM image; (c) EDS element mapping.

Figure S4: nano Cu catalyst after in-situ electrochemical reduction. (a) TEM image; (b) HR-TEM image; (c) EDS element mapping.

Figure S5: 5.88% A5-Cu catalyst after in-situ electrochemical reduction. (a) TEM image; (b) HR-TEM image; (c) EDS element mapping.

Figure S6: 11.11% A5-Cu catalyst after in-situ electrochemical reduction. (a) TEM image; (b) HR-TEM image; (c) EDS element mapping.

Figure S7: 20% A5-Cu catalyst after in-situ electrochemical reduction. (a) TEM image; (b) HR-TEM image; (c) EDS element mapping.

Figure S8. The XPS spectra of nano CuO and 13.04% A5-CuO catalyst. (a) Cu 2p; (b) N 1s.

Figure S9: The FE of others catalysts under different applied current density. (a) 5.88% A5-Cu; (b) 11.11% A5-Cu; (c) 20% A5-Cu.

Figure S10: The optimized partial current density of C_2+_ products in different A5 ionomer addition content (wt%) Cu catalysts.

Figure S11: The FE of HCOOH and CH_4_ for the Cu and different A5-Cu catalysts under the optimized reaction condition.

Figure S12: Chemical structure of different ionomers as mentioned in this document.

F

Figure S13: The FE of Sustainion and Fumion FAA-3 ionomer modified Cu catalysts. (a) 15.79% Sustainion-Cu; (b) 15.79% Fumion FAA-3-Cu.

Figure S14. (a) The H utilization efficiency of HER and C_2+_ products generation at 200 mA cm^-2^ for Cu and different ionomer modified Cu catalysts; (b) The *i-V* curves for Cu and different ionomer modified Cu catalysts.

Figure S15:15.79% Nafion-Cu catalyst after in-situ electrochemical reduction. (a) TEM image; (b) HR-TEM image; (c) EDS element mapping.

Figure S16: 15.79% A5-Cu catalyst after long-term experiment. (a) TEM image; (b) HR-TEM image; (c) EDS element mapping.

Figure S17: The FE of 15.79% A5-Cu catalyst in different concentration KHCO_3_ electrolyte. (a) 0.05 M; (b) 0.2 M; (c) 0.5 M.

Figure S18: The H_2_ formation rate of 15.79% A5-Cu catalyst in 1 M KHCO_3_ and 1 M KOH electrolyte.

Figure S19: The FE of 15.79% A5-Cu catalyst in different concentration KOH electrolyte. (a) 0.05 M; (b) 0.1 M; (c) 0.2 M; (d) 0.5 M.

Figure S20: The salt precipitation in CO_2_RR stability test of 15.79% A5-Cu catalyst at a constant current density of 200 mA cm^-2^ in MEA system with 0.1 M KOH as electrolyte.

Figure S21: (a) digital photo of the in-situ Raman setup; (b) digital photo of the in-situ ATR-SEIRAS setup.

Figure S22: Determination of the local CO_2_ concentration for Cu and different added content A5 modified Cu catalysts via electrochemical method using 1,4-benzoquinone as probe molecule. (a) bare Cu catalyst; (b) 5.88% A5-Cu catalyst; (c) 11.11% A5-Cu catalyst; (d) 15.79% A5-Cu catalyst; (e) 20% A5-Cu catalyst; (f) 15.79% Nafion-Cu catalyst.

Figure S23: The integral area of HCO_3_^-^ and CO_3_^2-^ species in the in-situ Raman spectroscopy. (a) bare Cu catalyst; (b) 15.79% A5-Cu catalyst.

Figure S24: The schematic diagram of K^+^ retention experiment.

Experiment details: K^+^ retention measurements were conducted in 0.1 M KHCO_3_ using a

three-electrode setup in a single cell. The Pt electrode was used as the counter electrode and a GDE (geometric area: 1 x 1 cm^2^) was used as the working electrode. After immersing the electrode into the electrolyte, a constant potential of -1.6 V (vs. Ag/AgCl) was applied for 200 s and then transferred to deionized water before applied the released potential. The concentration of K^+^ in the water was quantified by inductively coupled plasma optical emission spectrometer (ICP) detector as reported previously.^[6-7]^

Figure S25: (a) FEM simulations of OH^-^ concentration distributions vary as distance with or without ionomer; (b) FEM simulations of OH^-^ concentration curves vary as distance with or without ionomer.

Figure S26: The proportion of 2-HB-H_2_O over 5.88%, 15.79% and 20% A5-Cu catalysts at varying applied potentials during CO_2_RR.

Figure S27: (a) The FE of 5.88% A5-Cu catalyst under different applied current density in [K^+^] = 0.1 M KH_2_PO_4_/K_2_HPO_4_ buffer solution; (b) The *i-V* curves of 5.88% A5-Cu catalyst in 0.1 M KHCO_3_ and [K^+^] = 0.1 M KH_2_PO_4_/K_2_HPO_4_ buffer solution.

Figure S28: (a) The FE of 15.79% A5-Cu catalyst under different applied current density in [K^+^] = 0.1 M KH_2_PO_4_/K_2_HPO_4_ buffer solution; (b) The *i-V* curves of 15.79% A5-Cu catalyst in 0.1 M KHCO_3_ and [K^+^] = 0.1 M KH_2_PO_4_/K_2_HPO_4_ buffer solution.

Figure S29: (a) The FE of 20% A5-Cu catalyst under different applied current density in [K^+^] = 0.1 M KH_2_PO_4_/K_2_HPO_4_ buffer solution; (b) The *i-V* curves of 20% A5-Cu catalyst in 0.1 M KHCO_3_ and [K^+^] = 0.1 M KH_2_PO_4_/K_2_HPO_4_ buffer solution.

Figure S30: Equivalent circuit model used for EIS fitting.

Figure S31: EIS fitting results of catalysts at different potentials in 0.1 M KHCO_3_; (a) 5.88% A5-Cu; (b) 15.79% A5-Cu; (c) 20% A5-Cu.

Figure S32: The optimized adsorption models of intermediates on Cu(111) facet for H_2_O dissociation over 5.88% A5-Cu catalyst.

Figure S33: The optimized adsorption models of intermediates on Cu(111) facet for H_2_O dissociation over 15.79% A5-Cu catalyst.

Figure S34: The optimized adsorption models of intermediates on Cu(111) facet for H_2_O dissociation over 20% A5-Cu catalyst.

Figure S35: The optimized adsorption models of intermediates on Cu(111) facet for *CO converted to *OCCOH in C-C coupling step over 5.88% A5-Cu catalyst.

Figure S28. The optimized adsorption models of intermediates on Cu(111) facet for *CO converted to *OCCOH in C-C coupling step over 15.79% A5-Cu catalyst.

Figure S36: The optimized adsorption models of intermediates on Cu(111) facet for *CO converted to *OCCOH in C-C coupling step over 15.79% A5-Cu catalyst.

Figure S37: The optimized adsorption models of intermediates on Cu(111) facet for *CO converted to *OCCOH in C-C coupling step over 20% A5-Cu catalyst.

Figure S38: The *Δ*Gibbs free energy (*Δ*G) of three H_2_O dissociation calculation models.

Figure S39: The *Δ*Gibbs free energy (*Δ*G) of three C-C coupling calculation models.

Table S1: The preparation parameter of different ionomer-modification catalyst.

| Precursors | Catalysts* | CuO added in catalyst ink (mg) | Ionomer added in catalyst ink (mg) | Volumes of isopropanol (mL) (ρ=0.785 g cm^-3^) |
| --- | --- | --- | --- | --- |
| CuO | Cu | 720 | / | 103.2 |
| 4.76% A5-CuO | 5.88% A5-Cu | 720 | 36 | 94.9 |
| 9.09% A5-CuO | 11.11% A5-Cu | 720 | 72 | 99.1 |
| 13.04% A5-CuO | 15.79% A5-Cu | 720 | 108 | 103.2 |
| 16.67% A5-CuO | 20% A5-Cu | 720 | 144 | 107.3 |
| 13.04% Nafion-CuO | 15.79% Nafion-Cu | 720 | 108 | 101.8 |

*: After in-situ electrochemical activated, the ionomer mass ration was calculated using the following formula.

Mass ration = $\frac{m_{ion}}{{m_{Cu}+m}_{ion}}$ $\times100\%$= $\frac{m_{ion}}{720 \times0.8+m_{ion}}$ $\times100\%$

Table S2 The performance comparison of this work with the literature.

| Catalysts | *E* (V vs. RHE) | FE_C2+_ (%) | *I*_C2+_ (mA) | References |
| --- | --- | --- | --- | --- |
| 15.79% A5-Cu | -3.23* | 82 | 1025 | This work |
| Dual-Phase Cu | ~ | 81 | 322 | [8] |
| Al-doped Cu | -1.00 | 84.5 | 354.2 | [9] |
| 3D Cu-CS-GDL | -0.87 | 88.2 | 793.8 | [10] |
| 0.88% Pd-Cu | -0.88 | 80.8 | 646.4 | [11] |
| Cu NC/Cu-BTC | ~-1.65 | 92.3 | 276.9 | [12] |
| P-Cu_2_O-240 | ~ | 75.3 | 800 | [13] |
| High-CN Pot-Cu | -1.27 | 82.5 | 514.3 | [14] |
| AgCu-SAA | ~1.23 | 83.4 | 750.6 | [15] |
| PTFE/Cu/PVAQA_54_ | -0.94 | 91.2 | 273.6 | [16] |
| Ag/Cu | -1.80 | 89.2 | 553.9 | [17] |
| PAzPy/Cu | ~ | 84 | 168 | [18] |
| Cu-F-1 | -0.65 | 80 | 550 | [19] |
| 3D-CuAg-GDE | -0.88 | 82.04 | 565.18 | [20] |

Note: *Cell voltage.

Table S3: Determination of the local CO_2_ concentration for Cu and different added content A5 modified Cu catalysts via electrochemical method using 1,4-benzoquinone as probe molecule.

| Precursors | Catalysts | E_1/2_ in N_2_ (V vs. Ag/Ag^+^) | E_1/2_ in CO_2_ (V vs. Ag/Ag^+^) | *Δ*E (mV) | c(CO_2_)_A5-Cu_ /c(CO_2_)_Cu_ |
| --- | --- | --- | --- | --- | --- |
| CuO | Cu | -0.928 | -0.898 | 30 | 1 |
| 4.76% A5-CuO | 5.88% A5-Cu | -0.928 | -0.892 | 36 | 1.60 |
| 9.09% A5-CuO | 11.11% A5-Cu | -0.927 | -0.883 | 44 | 2.98 |
| 13.04% A5-CuO | 15.79% A5-Cu | -0.928 | -0.880 | 48 | 4.06 |
| 16.67% A5-CuO | 20% A5-Cu | -0.928 | -0.886 | 42 | 2.55 |
| 13.04% Nafion-CuO | 15.79% Nafion-Cu | -0.928 | -0.876 | 52 | 5.55 |

Note: To determine the local CO_2_ concentration for Cu and A5-modified Cu catalysts, 5 mM 1,4-benzoquinone was used as redox electrochemical probe molecule with 0.1 M [Bu_4_N][PF_6_]/DMF as electrolyte. The local CO_2_ concentration can be calculated by the following equation reported in the previous research.^[21]^

$$\frac{c \left( CO2 \right)1}{c \left( CO2 \right)2}= \frac{[e^{(\frac{\Delta E2}{12.84})}]}{[e^{(\frac{\Delta E1}{12.84})}]}$$

Table S4: The integral area of HCO_3_^-^ and CO_3_^2-^ species with applied potentials in the in-situ Raman spectroscopy.

| E (V vs. RHE) | | -0.2 | -0.4 | -0.6 | -0.8 | -1.0 | -1.2 | -1.4 | -1.6 | -1.8 |
| --- | --- | --- | --- | --- | --- | --- | --- | --- | --- | --- |
| Bare Cu | HCO_3_^-^ | 2568.6 | 2286.5 | 2105.3 | 2048.1 | 1831.1 | 1593.0 | 1281.1 | 1069.1 | 448.9 |
|  | CO_3_^2-^ | / | / | 321.7 | 558.7 | 706.0 | 840.3 | 995.3 | 1071.2 | 1185.5 |
| 15.79% A5-Cu | HCO_3_^-^ | 1174.2 | 832.0 | 606.0 | 841.7 | 544.4 | 437.4 | 544.5 | 42.0 | / |
|  | CO_3_^2-^ | 565.9 | 618.1 | 739.2 | 875.6 | 1092.9 | 1256.2 | 1320.6 | 1286.7 | 1266.7 |

**References**

[1] G. Kresse, J. Furthmüller, *Comput. Mater. Sci.* **1996**, *6*, 15-50.

[2] G. Kresse, J. Furthmüller, *Phys. Rev. B* **1996**, *54*, 11169-11186.

[3] J. P. Perdew, K. Burke, M. Ernzerhof, *Phys. Rev. Lett.* **1996**, *77*, 3865-3868.

[4] G. Kresse, D. Joubert, *Phys. Rev. B* **1999**, *59*, 1758-1775.

[5] P. E. Blöchl, *Phys. Rev. B* **1994**, *50*, 17953-17979.

[6] A. Ozden, J. Li, S. Kandambeth, X.-Y. Li, S. Liu, O. Shekhah, P. Ou, Y. Zou Finfrock, Y.-K. Wang, T. Alkayyali, F. Pelayo García de Arquer, V. S. Kale, P. M. Bhatt, A. H. Ip, M. Eddaoudi, E. H. Sargent, D. Sinton, *Nat. Energy* **2023**, *8*, 179-190.

[7] M. Fang, X. Miao, Z. Huang, M. Wang, X. Feng, Z. Wang, Y. Zhu, L. Dai, L. Jiang, *J. Am. Chem. Soc.* **2024**, *146*, 27060-27069.

[8] P. P. Yang, X. L. Zhang, P. Liu, D. J. Kelly, Z. Z. Niu, Y. Kong, L. Shi, Y. R. Zheng, M. H. Fan, H. J. Wang, M. R. Gao, *J. Am. Chem. Soc.* **2023**, *145*, 8714-8725.

[9] L. Zhang, J. Feng, L. Wu, X. Ma, X. Song, S. Jia, X. Tan, X. Jin, Q. Zhu, X. Kang, J. Ma, Q. Qian, L. Zheng, X. Sun, B. Han, *J. Am. Chem. Soc.* **2023**, *145*, 21945-21954.

[10] J. Bi, P. Li, J. Liu, S. Jia, Y. Wang, Q. Zhu, Z. Liu, B. Han, *Nat. Commun.* **2023**, *14*, 2823.

[11] D. Zhou, C. Chen, Y. Zhang, M. Wang, S. Han, X. Dong, T. Yao, S. Jia, M. He, H. Wu, B. Han, *Angew. Chem. Int. Ed.* **2024**, *63*, e202400439.

[12] X. Cao, S. Ren, X. Zhang, Q. Fan, Q. Chen, J. Yang, J. Mao, *Chem* **2024**, *10*, 2089-2102.

[13] Q. Geng, L. Fan, H. Chen, C. Zhang, Z. Xu, Y. Tian, C. Yu, L. Kang, Y. Yamauchi, C. Li, L. Jiang, *J. Am. Chem. Soc.* **2024**, *146*, 10599-10607.

[14] J. Jiao, X. Kang, J. Yang, S. Jia, Y. Peng, S. Liu, C. Chen, X. Xing, M. He, H. Wu, B. Han, *J. Am. Chem. Soc.* **2024**, *146*, 15917-15925.

[15] M. Wang, M. Fang, Y. Liu, C. Chen, Y. Zhang, S. Jia, H. Wu, M. He, B. Han, *J. Am. Chem. Soc.* **2025**, *147*, 16450-16458.

[16] J. Cheng, L. Chen, Y. Zhang, M. Wang, Z. Zheng, L. Jiang, Z. Deng, Z. Wei, M. Ma, L. Xiong, W. Hua, D. Song, W. Huo, Y. Lian, W. Yang, F. Lyu, Y. Jiao, Y. Peng, *Nat. Commun.* **2025**, *16*, 3743.

[17] Z. Zhang, X. Ma, Y. Song, X. Yang, Q. Fang, Y. Yamauchi, J. Tang, *Angew. Chem. Int. Ed.* **2025**, *64*, e202511704.

[18] X. Chai, P. Shi, J. Zhao, S. Huang, K. Jiang, C. Lu, L. He, J. Chen, T. Wang, X. Zhuang, *Angew. Chem. Int. Ed.* **2025**, *64*, e202513306.

[19] L. Liu, Y. Li, K. A. Chiaw, S. Xi, S. Cheng, S. U. Dighe, H. Chang, B. Y. Tay, M. Tan, K. W. Huang, L. Lai, Y. Ou, L. Zhang, *Angew. Chem. Int. Ed.* **2025**, *64*, e202506184.

[20] G. Xie, Z. Zhu, D. Liu, W. Gao, Q. Gong, W. Dong, Y. Zhai, W. Guo, X. Sun, *Angew. Chem. Int. Ed.* **2025**, *64*, e202510167.

[21] X.-R. Qin, J.-J. Li, L.-L. Wang, H. Liu, Z.-T. Yang, G.-J. Feng, X.-R. Wang, X.-X. Cheng, C. Zhang, Z.-Y. Yu, T.-B. Lu, *Nat. Commun.* **2025**, *16*, 4417.
